# Preparation and Characterization of Coating Based on Protein Nanofibers and Polyphenol and Application for Salted Duck Egg Yolks

**DOI:** 10.3390/foods9040449

**Published:** 2020-04-07

**Authors:** Qiannan Wang, Weihua Liu, Bo Tian, Dongmei Li, Chunhong Liu, Bin Jiang, Zhibiao Feng

**Affiliations:** 1 Department of Applied Chemistry, Northeast Agricultural University, Harbin 150030, China; W18346111097@163.com (Q.W.); 15561851295@163.com (W.L.); lidongmei@neau.edu.cn (D.L.); liuchunhong@neau.edu.cn (C.L.); 2 College of Food Science, Northeast Agricultural University, Harbin 150030, China; tianbot@163.com

**Keywords:** whey protein isolate nanofibrils, edible coating, antibacterial activity, salted duck egg yolks

## Abstract

Salted duck egg yolk (SDEY) is one of the traditional pickled egg products in Asian countries, which suffers from the weight loss and deterioration of texture characteristics during storage. To better maintain the texture of SDEY, an edible coating based on whey protein isolate nanofibers (WPNFs) with glycerol (Gly) as a plasticizer and incorporating carvacrol (CA) as an antimicrobial agent was developed. Whey protein isolate (WPI, 5%) was used to self-assemble into WPNFs at 80 °C for 10 h. The particle size, zeta-potential and microstructure of WPNFs–CA emulsion were investigated to evaluate the distribution. Results proved that WPNFs–CA emulsion had smaller particle size and better distribution than WPI–CA emulsion. WPNFs–CA/Gly edible coating was then prepared based on WPNFs–CA emulsion. The WPNFs–CA/Gly edible coating exhibited higher antibacterial activity while the WPNFs–CA/Gly film had smooth and continuous surfaces and better transmittance compared with other samples. Furthermore, weight losses and textural properties changes of SDEYs with WPNFs–CA/Gly coating were evaluated. Results proved that salted duck egg yolks with WPNFs–CA/Gly coating exhibited lower weight losses. Textural properties were significantly improved by the WPNFs–CA/Gly coating on SDEYs than those uncoated samples. It was noted that the egg yolks coated with the WPNFs–CA/Gly coating had the lowest hardness increase rate (18.22%). Hence, WPNF-based coatings may have a good development prospect in the food industry.

## 1. Introduction

Edible coating (EC) is a thin layer of edible material formed by a coating on food. Generally, ECs are applied to the surface of the food by means of roll coating or dipping in the form of a liquid, thereby protecting the food. ECs can also be prepared into edible films (EFs). ECs and EFs are widely used for the protection of food quality, thereby extending the shelf life of perishable food, especially those susceptible to oxidative and microbiological deterioration [1]. In recent years, people have become more and more interested in the preparation of nanostructures and their application in the food field. Nano ECs are considered to be a new packaging material with gas barrier properties [2].

Whey protein isolate (WPI) is a by-product of the cheese industry and casein, with high nutritional and functional values [3,4]. Compared with polysaccharides and lipid membranes, WPI can be made into a transparent film that blocks gases, aromas, and oils [5]. However, the comprehensive performance of pure protein films is unsatisfactory. Therefore, it is necessary to improve the comprehensive performance of protein film through various methods. For example, WPI is processed by physical, chemical, or enzymatic methods in industrial practice. In chemical methods, especially at isoelectric points or lower pH, WPI can form more stable three-dimensional network structures [6], which are called whey protein isolate nanofibers (WPNFs). Normally, the protein is hydrolyzed to form a polypeptide, and the fibers are made up of polypeptides. Peptides are the basic units of WPNFs, and the *β*-sheet structure is an important secondary structure of WPNFs [7]. Positive potential is an important factor in inducing the formation of protein nanofibers [8]. The formation of WPNFs is an equilibrium state. Fiber aggregates are formed by the mutual aggregation of peptides, and this aggregation is mutually repelled by the positive surface potential, thereby preventing the occurrence of transitional aggregation. Therefore, a strong acid condition is a key factor information and stability of fibers. Due to their special structures and physicochemical properties, WPNFs are used in a variety of food fields. They can be used as geling agents, foaming agents, emulsifiers, or biological carriers, as well as a packaging material [9]. In emulsions, the fibrils can also adsorb on the oil/water interface to form a coating around the oil droplets [10]. This has a beneficial effect on the stability of the emulsions, which indicates the high potential of WPNFs in the food industry.

Salted duck egg yolk (SDEY) is one of the traditional pickled egg products in Asian countries. Eggs can offer nutrients with great biological value such as vitamins, minerals, lipids, and proteins for human growth and good health [11]. Due to the unique flavor and rich nutrients, SDEY is widely used in the processing of baked goods, such as moon cake, egg yolk cake, and zongzi (traditional Chinese rice-pudding) [12,13]. The main factors which directly associated with egg yolk texture deterioration are water migration, the Maillard reaction, and fat oxidation during storage, which may lead to the hardening of the yolk surface. Hardening is considered as a challenge for storage, which greatly limits SDEYs’ shelf life, which in turn affects the color and taste of such products. At present, the studies on SDEYs are mainly focused on the processing method [14], but there are few studies on the preparation and application of WPNFs-based edible coating to solve the hardening of egg yolks [15].

For the purpose of better maintaining the texture of SDEY, a novel nano-edible coating based on WPNFs, containing carvacrol (CA) and glycerol (Gly), was developed. It was proved by several key technologies that edible coatings prepared with functionalized WPNFs could be considered a diverse tool for the long-term preservation of food. Hence, WPNFs-based coatings might have a good development prospect in the food industry.

## 2. Materials and Methods

### 2.1. Materials

WPI (protein content >91.5%) was acquired from Hilmar Industries (Hilmar, CA, USA). Carvacrol (purity >99.9%) was obtained from Aladdin Reagent Co. (Shanghai, China). Pathogenic bacteria were obtained from BNCC Biological Technology Co. Ltd. (Nanjing, China). Salted duck eggs were purchased from the local market (Harbin, China).

### 2.2. Whey Protein Isolates Nanofiber (WPNF) Formation

WPI solution (5%, *w/v*) was prepared by dissolving WPI in deionized water. Then, 3 M HCl was carefully added to readjust the pH of the solution to 2.0 at room temperature. The solution was stirred at room temperature for 30 min. Then the solution was centrifuged at 9000× *g* for 15 min at 4 °C (Z236HK Hermle, Wehingen, Germany). To remove undissolved proteins, the above supernatant was vacuum-filtered through a 0.45-µm pore size fiber membrane (Aladdin, Shanghai, China). The filtered solution was incubated at 80 °C for 10 h with 220 rpm constant magnetic stirring to obtain WPNFs solution. An aliquot of the filtered solution (WPI-c) was preserved to be used as a control in the following experiments.

WPNFs were respectively treated by including ultrasonication, shear homogenization, and high-pressure homogenization, and then the effects of these three physical methods on WPNFs morphology were compared. The WPNF samples were homogenized by shear homogenization with 10,000 rpm at 5 min (ESB-500, ELE Company, Shanghai, China). The high-pressure homogenization was processed at 40 MPa with four passes using a high-pressure homogenization (Panda 2 K; Niro Soavi Deutschland, Lübeck, Germany). Ultrasonication was performed by using an ultrasonic cell grinder with 400 W for 5 min (JY92-2D, Scientz Company, Ningbo, China).

#### 2.2.1. Transmission Electron Microscopy (TEM)

Above WPNFs samples were ultrafiltrated using previously reported methods to reduce the background in TEM images [8]. Briefly, the WPNFs samples were diluted ten-fold with hydrochloric acid solution (pH = 2.0). Then the samples were transferred to a special copper mesh on a carbon film, stood for 15 min, and contacted with filter paper to aspirate excess samples. A droplet of uranyl acetate (2%) was then added to the dried copper and stood for 8 min. Electron micrographs of the above samples were taken using TEM (H-7650 transmission electron microscope, Hitachi, Tokyo, Japan).

#### 2.2.2. Sodium Dodecyl Sulfate–Polyacrylamide Gel Electrophoresis (SDS–PAGE)

SDS–PAGE was carried out as described by Jiang et al. [16] and Oboroceanu et al. [17]. The samples were taken from the bath at the prescribed point-in-time (0, 1, 2, 3, 5, 7, 9, and 10 h) and immediately cooled on ice. The samples were mixed with reducing sample buffer, which was composed of 2% SDS, 0.622 M Tris-HCl (pH = 6.8), 20% glycerol, and 5% *β*-mercaptoethanol), with a ratio of 1:14 (sample: buffer). The separation process was performed under reducing conditions at a constant voltage of 150 V for 12% polyacrylamide gels in a Mini Protean II system (Bio-Rad, Guangzhou, China). Then, 0.5% Coomassie Brilliant Blue R-250 (Sigma-Aldrich, St. Louis, MO, USA) was used to stain.

### 2.3. Preparation of Emulsions

High-pressure homogenization was used for the preparation of emulsion. For emulsification, 0.1 g CA was dissolved in 19.9 g WPNFs solution obtained in Section 2.2 and treated by shear homogenization at 5000 rpm for 1 min. Homogenizing was performed for emulsion at 40 MPa with four passes by a high-pressure homogenization (Panda 2 K; NiroSoavi Deutschland, Lübeck, Germany). The obtained emulsions (WPI–CA and WPNFs–CA) were stored at 4 °C for subsequent analyses. The WPI-c and WPNFs solutions treated in the same way were used as controls. The WPI-c treated with four passes by a high-pressure homogenization was defined as a WPI solution.

#### 2.3.1. Measurement of Particle Size and Zeta-Potential

The particle sizes of different samples, including WPI solution, WPNFs solution, WPI–CA emulsion, WPNFs–CA emulsion, were measured at pH 2.0, using dynamic light scattering (Zetasizer Nano system, Malvern Instruments Inc., Malvern, UK) [18]. All samples were diluted ten-fold with hydrochloric acid solution (pH 2.0).

Direction and velocity that the droplets moved in the applied electric field was measured to evaluate zeta-potential. The zeta-potential of WPI solution, WPNFs solution, WPI–CA emulsion, WPNFs–CA emulsion was measured by a Zetasizer Nano system (Malvern Instruments Inc., Malvern, UK). All of the specimens were diluted ten-fold with hydrochloric acid (pH 2.0) before the zeta-potential measurements prior to analysis [19].

#### 2.3.2. Confocal Laser Scanning Microscopy (CLSM)

Confocal laser scanning microscopy (Model IX3-CBH, OLYMPUS, Tokyo, Japan) was used for further observation of the emulsion droplet microstructure. The samples were diluted approximately 50-fold with hydrochloric acid (pH 2.0). Distilled water was used for all the measurements. Briefly, 0.1% (*w/v*) Nile red and 0.1% (*w/v*) Nile blue in isopropyl alcohol were used for staining of the oil phase and the aqueous phase (protein), respectively. The above emulsions (1 mL) were stained with a mixed fluorescent dye solution (40 μL) consisting of Nile red and Nile blue. Next, 2 μL stained emulsions were placed on concave slides and covered with coverslips. Nile red and Nile blue were excited at 488 nm with an argon laser for Nile red and at 633 nm with a helium neon laser, respectively.

### 2.4. Preparation and Functional Properties of Edible Coatings (ECs)

To obtain ECs, Gly 5% (*w/v*) as a plasticizer was added to above mentioned WPNFs–CA emulsions. After being stirred for 30 min at 90 °C, the solutions were cooled to room temperature. Also, the WPI solution, WPNFs solution and WPI–CA emulsion were produced with the same procedure and used as a control. The obtained coatings were named as WPNFs–CA/Gly, WPI/Gly, WPNFs/Gly, WPI–CA/Gly coatings, respectively. Then, 20 mL of the degassed ECs were added into the Teflon-coated plates that were employed as supports to prepare the films, which were placed on surface inside an environmental chamber (Blue-Pard Pharma, China) at 60 °C for 18 h. The obtained films were named as WPNFs–CA/Gly, WPI/Gly, WPNFs/Gly, WPI–CA/Gly films, respectively.

#### 2.4.1. Antibacterial Activity Analysis

*Listeria monocytogenes* (CMCC 54004), *Salmonella Enteritidis* (CMCC 50071), *Staphylococcus aureus* (CMCC 26112), and *Escherichia coli* (CMCC 44113) were used for antibacterial activity analysis of ECs. Antibacterial activities were determined by the agar disk diffusion method. Then, 100 µL bacteria suspension, which contained 10^7^ colony-forming units per mL (CFU/mL), was smeared on the surface of a Mueller–Hinton agar plate (Aladdin Reagent Co., Shanghai, China). A 10-mm diameter sterile paper disc which was impregnated with 1 mL edible coating emulsion was positioned on the center of the inoculated plate. The agar plates were incubated at 37 °C for 24 h. A caliper (Deli Co., Ningbo, Zhejiang, China) was used to determine the diameters (mm) of the inhibition zones.

#### 2.4.2. Scanning Electron Microscopy (SEM)

The cross-section of the films was examined by SEM (Hitachi S-3400N). The films were coated with a fine gold layer before obtaining the micrographs [20]. All films were examined using an accelerating beam at a voltage of 5 kV.

#### 2.4.3. Determination of Physical Properties of Composite Films

Thickness of film was measured by digital external micrometer (Mitutoyo Co., Kawasaki, Japan) and the overall thickness was expressed as an average generated randomly from each film at 15 different points.

The transmittance (%) of the films was measured by a Shimadzu UV-2500 spectrophotometer (Tokyo, Japan). Film was cut into 1 × 4 cm rectangular pieces. Then the cut films were fixed to one side of a spectrophotometer cell. An empty cell was used as control. Three duplications were performed for each film to guarantee the accuracy. The transparency of sample was measured at 600 nm. Relative transparency, which was an approximation, was calculated according to Formula (1):

(1) $$\mathrm{Transmittance}\;(\%)=(\frac{T_{600}}{d})\;\times \;100\;$$

where *T*_600_ is transparency of the film at 600 nm; *d* is the averaged thickness (mm).

A Minolta colorimeter (Cr 410, Konica Minolta, Tokyo, Japan) was used to measure the color parameters of the films with a standard white plate applied for calibration. The results were expressed according to the CieLab color system, where L* was 0 for black and 100 for white, a* represented red (+) to green (−), and b* values indicated yellow (+) to blue (−). The mean values were then calculated.

### 2.5. Quality Assessment of Salted Duck Egg Yolks (SDEYs)

#### 2.5.1. Pretreatment of Salted Duck Egg Yolks (SDEYs)

After being broken manually, the eggs were put into a separator to separate yolk from albumin. Albumen that was adhered to the vitellin membrane was removed by careful rolling on filter paper (Whatman). After being soaked in the edible coating emulsion for 1 min, yolks were naturally drained at room temperature for 30 min. Coated samples and uncoated SDEYs (control samples) were stored at 4 °C for 10 days.

#### 2.5.2. Weight Loss

All SDEYs samples were weighed every two days during the storage period. The weight loss (*W*_1_) was determined by Formula (2):

(2) $$W_{l}\;\left(\%\right)=\left(\frac{W_{1}{\;-\;W}_{2}}{W_{2}}\right)\;\times \;10$$

where *W_1_* is the initial weight and *W*_2_ is the SDEYs weight measured at different storage time points.

#### 2.5.3. Evaluation of Physicochemical Properties of Coated SDEYs

Texture profile analysis (TPA) of SDEYs, including hardness, springiness, and chewiness, was performed by a texture analyzer (TA-TX2, Stable Micro Systems, Goldaming, UK). The test parameters were the 36R probe with two compression-decompression cycles, 5.0 mm/s of pre-speed and post-speed, 1 mm/s of test speed, 20% of the distance, and a trigger force of 5 g. The samples were tested at 25 °C.

### 2.6. Statistical Analysis

All treatments were repeated three times. The data were expressed as mean values of triplicates with the standard deviation (error bars). SPSS (20.0) software (Chicago, IL, USA) was used for statistical analysis. Significant differences (*p* < 0.05) between means were evaluated by Duncan’s multiple range test.

## 3. Results

### 3.1. Transmission Electron Microscopy (TEM) Micrographs of WPNFs

**Figure 1:**
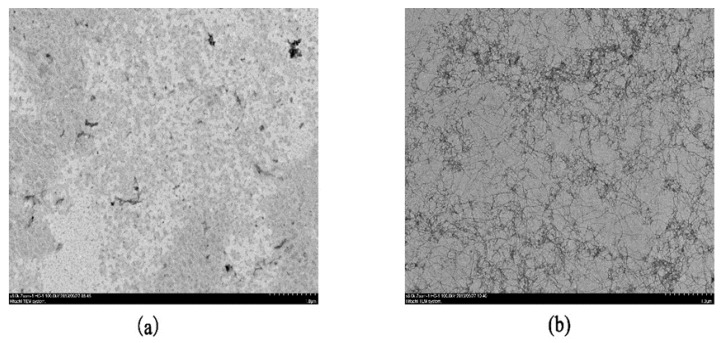
TEM micrographs (negatively stained) of different samples. (**a**) Whey protein isolate filtered solution (WPI-c); (**b**) whey protein isolate nanofibers (WPNFs; prepared by heating 5% WPI at 80 °C and pH 2.0 for 10 h).

TEM is a common method to observe the micro-morphology of fibrils. The TEM images of the WPNFs and WPI-c are showed in Figure 1. WPI-c was heated after 10 h, the shapes of the protein were changed from spherical particles (Figure 1a) to filamentous structures (Figure 1b). The protein was hydrolyzed to form a polypeptide at a high temperature of 80 °C, causing the partial structure of the globular protein to unfold, and the hydrophobic group previously in the structure was exposed. The disulfide bond between different cysteine molecules might cause aggregation of cysteine molecules in polypeptides, thereby causing fibrillar aggregation. As indicated in Figure 1b, heat treatment on the WPI-c at pH 2.0 resulted in the formation of fibrillar aggregates with a nanometric diameter and micrometric length, which is in accordance with previous studies [21]. WPNFs were slender and unbranched, and the windings were intertwined to form a network structure. This high ratio of the length versus the diameter of the fibrils makes fibrils promising candidates for using fibrils as a structural substance in foods. There has been research showing that, with the addition of WPNFs, the viscosity, gel strength [10] and foaming [8] of solutions were increased.

**Figure 2:**
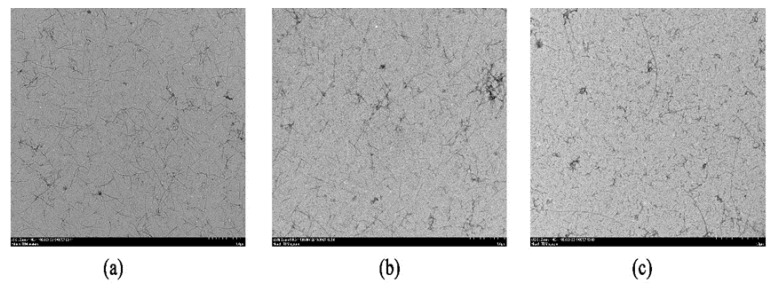
TEM micrographs (negatively stained method) of WPNFs treated by different physical methods: (**a**) WPNFs treated by shear homogenization, (**b**) WPNFs treated by ultrasonication, (**c**) WPNFs treated by high-pressure homogenization.

A previous study has demonstrated that particle property, especially the shape, has a strong influence on the stability and properties of emulsions [22]. The length of nanofibrils from food proteins affects their emulsifying properties. As can be seen from Figure 1b, WPNFs with a length of several micrometers were formed, consistent with previous reports [9,21]. Figure 2 shows the TEM micrographs of WPNFs treated by different physical methods (shear homogenization, ultrasound, and high-pressure homogenization, respectively). The length of the fibers treated by the three physical methods was shorter than the length of the untreated fibers. For this reason, all three methods led to the shortening of the fibril length. However, there were differences among the length of the WPNFs after different physical treatment methods. Fibril treated by high-pressure homogenization showed the shortest length. It has been reported that the length of the fibrils was closely related to the stability of the emulsion [23]. With respect to the stabilization of interfaces in emulsions, short fibrils, compared to long fibrils, might lead to better covering efficiency around small oil droplets. Moreover, short fibrils might prevent the occurrence of bridging flocculation in fibril-stabilized emulsions [24]. Therefore, there was a bold conjecture that the fibril treated by high-pressure homogenization could stabilize the emulsion. Therefore, the high-pressure homogenization method was used to treat the solution preparing the emulsion.

### 3.2. SDS–PAGE

**Figure 3:**
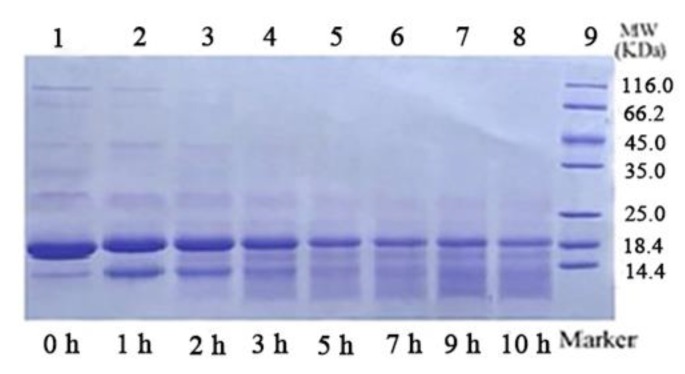
SDS–PAGE of 5% WPI-c at pH 2.0, heated at 80 °C for various heating times; Lanes 1–8 represent WPI-c heated for 0, 1, 2, 3, 5, 7, 9, 10 h, respectively; Lane 9, polypeptide molecular-weight marker.

Figure 3 shows the reducing-mode SDS–PAGE image of samples. A major broad band around 18 kDa was observed in Lane 1, which corresponds to the *β*-lactoglobulin (*β*-lg) monomeric form. The degradation of proteins under heating at acidic conditions is time-dependent. With the prolongation of heating time, WPI degraded to low molecular weight peptides, resulting in a progressive decrease of the corresponding electrophoretic band. TEM results (Figure 1) proved that WPNFs with a length of several micrometers were formed after WPI-c was heated for 10 h. However, unstained proteinaceous material was not appeared at the top of the stacking gel or within the wells, and only low-molecular-weight protein fragments were seen in Lane 8. It indicated that fibrils had been dissociated by the reducing sample buffer, and WPNFs were composed of peptide fragments [17]. These observations appeared to consistent previous studies that *β*-lg was hydrolyzed into peptides with molecular weights less than 10 kDa at pH 2 and 80 °C for 10 h to form fibrils [25].

### 3.3. Properties of WPNF-Based Emulsions

**Figure 4:**
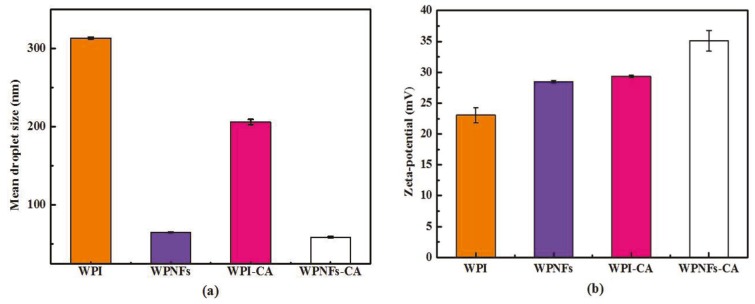
Mean size (**a**) and zeta-potential (**b**) of WPI solution, WPNFs solution, WPI– carvacrol (CA) emulsion and WPNFs–CA emulsion, respectively.

The droplet size distribution of emulsions is a key factor for estimating the emulsifying effect and emulsion stability [26]. The droplet sizes for emulsions mentioned above were measured and the results are shown in Figure 4a. Compared with WPI and WPNFs solutions, WPI–CA and WPNFs–CA emulsions showed smaller mean size. The mean size of WPNFs-based emulsions decreased from 65.0 to 58.9 nm with the addition of CA substances. The mean size of the WPI solution was around 313.6 nm, followed by 205.9 nm for WPI-CA emulsion. The results revealed that WPNFs solution and WPNFs–CA emulsions have a relatively uniform distribution. The reason was WPNFs with micrometric length and nanometric diameter have high aspect ratios (length/diameter) [27]. The control over the emulsion stability was closely linked to the surface rheology and could be affected by aspect ratio and surface coverage. WPNFs are polypeptide aggregates [25]. After homogenization by high pressure, the intermolecular forces may be destroyed, which leads to a decrease in particle size of WPNFs compared to the WPI solution. In addition, non-spherical particles can be used to efficiently form stable emulsions [22]. As mentioned previously, the decrease in droplet diameter of WPNFs–CA emulsion in comparison to WPI–CA emulsion could be related to the effectiveness of droplets’ break up during high-pressure homogenization due to appropriate viscosity ratio of dispersement to an aqueous phase.

The zeta-potential values of WPI solution, WPNFs solution, WPI–CA emulsion, and WPNFs–CA emulsion are depicted in Figure 4b. The zeta-potential values of WPI emulsion were around 23.0 mV, followed by 28.5 mV for WPNFs emulsion, 29.3 mV for WPI–CA and 35.1 mV for WPNFs–CA emulsion. The zeta-potential measurements demonstrated that the emulsion stabilized by WPNFs–CA are strongly positive charged. Generally, zeta-potential values depend on the charge on the actual particle and also the charge relevant to cationic and anionic ions that move with the particle in the electric field. It should also be mentioned that zeta potential values of the WPNFs–CA emulsion were highly positive, which could stabilize emulsion against flocculation through electrostatic repulsion [28].

### 3.4. Emulsion Microstructure

**Figure 5:**
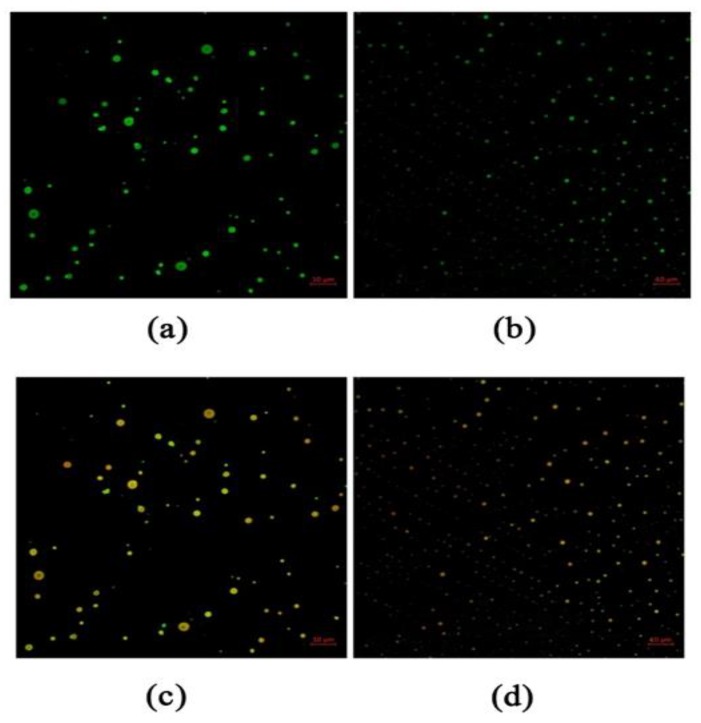
Confocal laser scanning microscopy (CLSM) images of samples (**a**) WPI was stained with Nile blue, (**b**) WPNFs was stained with Nile blue, (**c**) WPI was stained with Nile blue and carvacrol (CA) was stained with Nile red, (**d**) WPNFs was stained with Nile blue and carvacrol (CA) was stained with Nile red. Microscopic images were obtained in the overlap fluorescence field. The fluorescent dyes were simultaneously excited at 488 nm for Nile red (red) and 633 nm for Nile blue (green).

As shown in Figure 5, the microscopic images of the emulsions were analyzed by CLSM. To distinguish the aqueous and oil phases, proteins were stained with Nile Blue (marked in green) and CA was stained with Nile Red (labeled in red), while the emulsions droplets appeared orange on a dark background. WPI in the aqueous solution formed large soluble particles (Figure 5a), and WPNFs formed a small aggregation of droplets (Figure 5b) after high-pressure homogenization. It was consistent with the results of the average particle size of the above emulsion, which is shown in Figure 4a. Figure 5c,d indicates that WPI and WPNFs were combined with CA. Compared with WPI–CA emulsion, WPNFs–CA emulsion showed smaller average particle size and a better degree of binding, indicating that the formation of WPNFs was more conducive to the combination with the oil phase. This might be due to the better hydrophobicity of WPNFs, and the emulsification of WPNFs was related to the hydrophobicity of the molecules. Influenced by the distribution of hydrophobic groups in the molecule, the combination of WPNFs and oil was promoted, and the WPNFs were better adsorbed to the oil–water interface. The emulsion was formed into an oil-in-water system, where CA was the oil phase. The oil–water interface had a certain degree of strength and protected the dispersed droplets from collapsing when they collided with each other. When the two components were adsorbed on the interface, a “composite” was formed, and the alignment was tight. The interface film was a mixed film with a high strength influencing the stability of the emulsions.

### 3.5. Physical Properties of Edible Films (EFs)

The measured thicknesses of the EFs are shown in Table 1. Comparison with the control, the thickness of films with CA incorporated into increased (*p* < 0.05). It varied from 0.184 to 0.232 μm.

**Figure 6:**
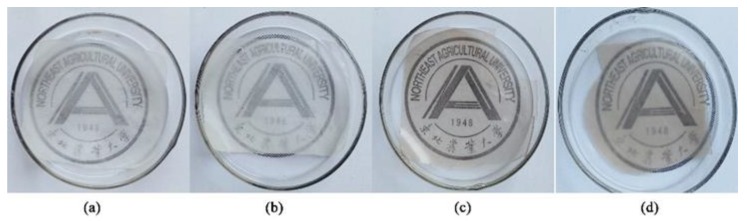
Effect diagrams of films transparency: (**a**) WPI/Gly, (**b**) WPI–CA/Gly, (**c**) WPNFs/Gly, (**d**) WPNFs–CA/Gly.

Optical properties of EFs, such as transmittance, opacity, and color values, are important properties that influence their appearance, acceptance, and suitability for various applications [1]. The transmittance of the EFs is shown in Table 1. With the same other components, the effect of the presence or absence of CA on the transmittance of films was compared. After essential oils were incorporated into the films, a lower transmission was obtained at 600 nm. The result suggested that CA effectively prevented the transmission at 600 nm. WPNFs-based films had higher transmittance (*p* < 0.05) (Figure 6). The reason was WPNFs-based films had a smooth and continuous surface, while the surface of WPI films was rough. This rough surface increased the opacity for a light-scattering effect as discussed in Section 3.6.2. In transparent visible material, non-uniformities and non-continuous in the composition of the material can bring about obvious changes in optical properties.

The color values of different films (WPI/Gly, WPI–CA/Gly, WPNFs/Gly, and WPNFs–CA/Gly films) are also shown in Table 1. Color values of the films were expressed as L*-(lightness/brightness), a*-(redness/greenness), and b*-(yellowness/blueness) values. Compared with the WPI/Gly and WPNFs/Gly films, L*-values of the WPI–CA/Gly and WPNFs–CA/Gly films were decreased, while b*-values of the WPI–CA /Gly and WPNFs–CA/Gly films were increased (*p* < 0.05). The b* (1.50) and a* (2.29) values of WPNFs/Gly film were significantly different from that of WPNFs–CA/Gly and control WPI-based films (*p* < 0.05). The tendency to yellowness was also verified in WPI-based films. The yellowness was due to the -NH_2_, which may interact with carbonyl groups (C=O) of lipid oxidation products which present as impurities in raw protein, via the Maillard reaction, during the drying of the films. The color of the WPNFs-based films was changed from light yellow to red. Because the WPI was undergoing the Maillard reaction during the heating process, the browning of the protein solution occurred. So the color of the WPNFs-based films was light red. Compared with the WPI-based films, the WPNFs-based films had better transparency.

### 3.6. Functional Properties of Edible Coating Emulsions (ECs) and Edible Films (EFs)

#### 3.6.1. Antibacterial Activity Analysis

**Figure 7:**
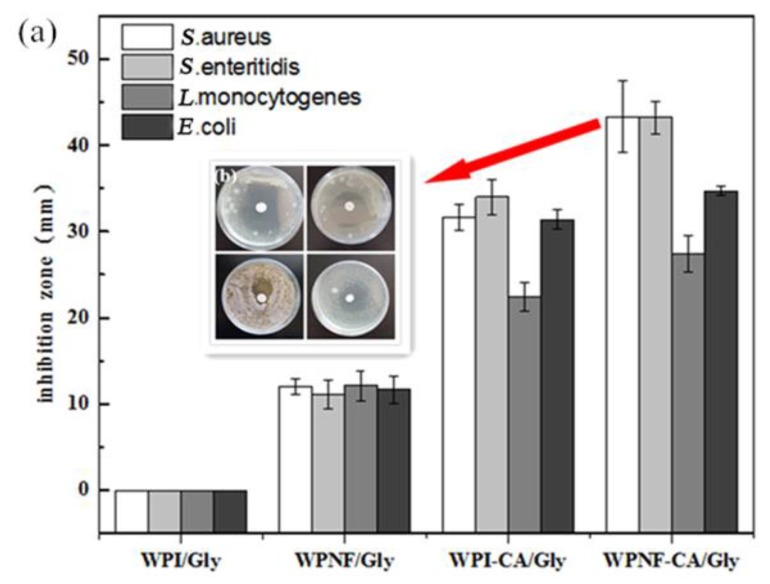
Antimicrobial activity of edible emulsions against *Listeria monocytogenes* (CMCC 54004), *Staphylococcus aureus* (CMCC 26112), *Salmonella enteritidis* (CMCC 50071), and *Escherichia coli* (CMCC 44113). (**a**) The inhibition zones of edible coatings; (**b**) representative picture of inhibitory zones of WPI/Gly and WPNFs–CA/Gly coatings. Error bars indicate the standard deviation of the mean.

The effectiveness of ECs on inhibiting *Listeria monocytogenes*, *Staphylococcus aureus*, *Salmonella enteritidis*, and *Escherichia coli* is demonstrated in Figure 7. In the present study, the WPI/Gly coating exhibited essentially no bacteriostatic activity. The WPNFs/Gly coating showed a slight antibacterial activity against tested bacteria, which was likely due to the antioxidant ability of the WPNFs [9,29]. It was due to the generation of bioactive peptides during fibril formation. When CA was incorporated into ECs, WPI–CA/Gly and WPNFs–CA/Gly coatings had greater inhibition zones due to the diffusion of bioactive compounds of CA. Microbicidal activity of CA may be attributed to the high electrophilic properties of the carbonyl group adjacent to the double bounds, which can activate CA to react with nucleophiles, such as the protein sulfhydryl and amino groups of the microorganism [30]. The antibacterial effect of CA was mainly achieved by destroying the integrity of the cell membrane and cell walls, and causing changes in cell membrane permeability and cell morphology [31]. Compared with the WPI–CA/Gly coating, the WPNFs–CA/Gly coating showed better antibacterial activity (44.33 ± 4.16, 43.23 ± 1.91, 27.43 ± 2.14, and 34.73 ± 0.46 mm for *S.aureus*, *S. enteritidis*, *L.monocytogenes* and *E.coli*, respectively). This may be due to the synergistic action of the antioxidant properties of the WPNFs with the bactericidal properties action of CA [32].

The action of essential oils against food spoilage organisms and food-borne pathogens is high [31]. Research showed oregano essential oils had the strong inhibitory effect on the growth of *E. coli*, *Listeria* *monocytogenes*, *Salmonella typhimurium,* and *Staphylococcus* *aureus*. Due to phenolic compounds monoterpene and p-cymene, oregano essential oils have powerful antimicrobial activity [30].

#### 3.6.2. SEM

**Figure 8:**
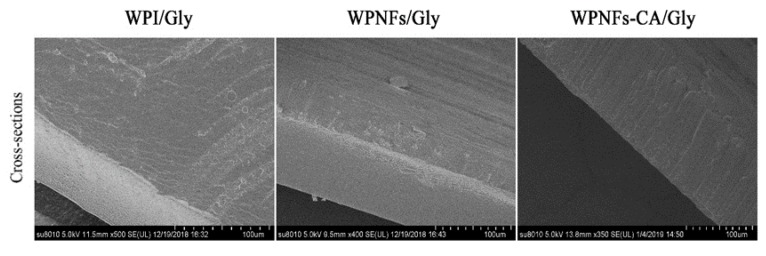
Scanning electron microscopy images of the cross-sections of edible films.

The cross-sections of scanning electron micrographs for WPI/Gly, WPNFs/Gly, and WPNFs–CA/Gly films are shown in Figure 8. It was evident from the micrographs that the WPI/Gly film had a rough cross-section, and was scattered with dispersed irregular particles, and had some pores. Conversely, since the WPNFs appeared to be inked by fine stands to form a continuous network, the cross-section of WPNFs/Gly film was relatively flat and continuous [33]. Due to this and the dense and continuous network structure, less pores remained while the WPNFs material formed a continuous and smooth matrix.

### 3.7. Functional Properties of the ECs on the Preservation of Salted Duck Yolk Eggs

#### 3.7.1. Weight Loss

Salted egg whites are a by-product of salted egg yolks [34]. As a result of moisture loss, the weight of SEDYs will be reduced, and this will cause adverse effects on both nutritional quality and product shelf life.

**Figure 9:**
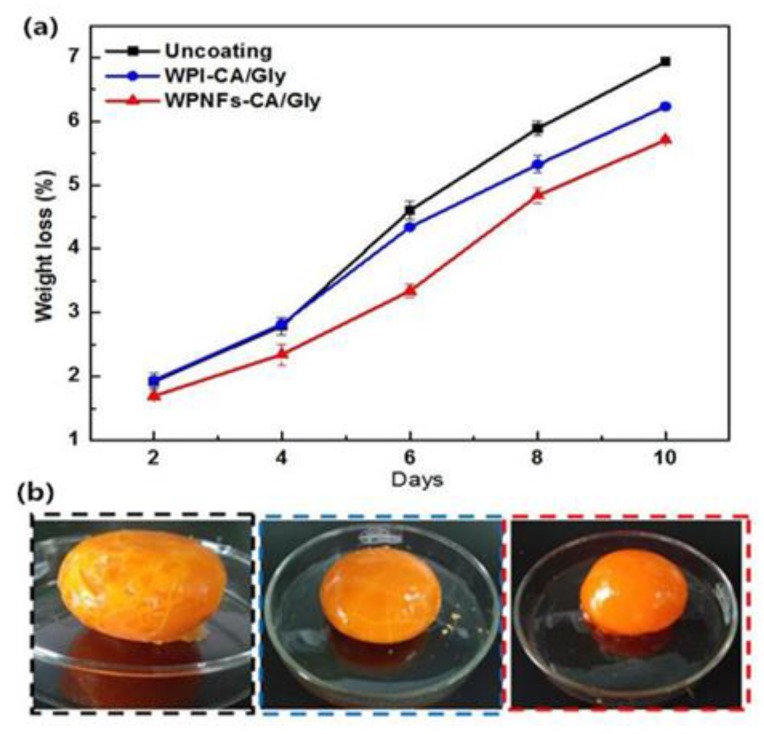
(**a**) Weight loss of coated and uncoated SEDYs during the storage periods for samples stored 10 days at 4 °C. (**b**) The actual picture of the SEDYs after 10 days of storage: the samples processing sequence were SEDYs uncoated, SEDYs coated with WPI–CA/Gly coating, SEDYs coated with WPNFs–CA/Gly coating.

Figure 9a shows the weight loss of the coated egg yolk stored at 4 °C for 10 days. With the extension of storage time, the weight loss rate of all experimental groups showed an upward trend. The weight loss of SEDYs coated with WPI/Gly and WPNFs–CA/Gly coatings was significantly lower than that of the uncoated salted egg yolk control group. Salted egg yolk coated with WPNFs–CA/Gly coating was superior to salted egg yolk treated with other composite membranes, showing the lowest weight loss. What is worth mentioning is the most significant difference between the weight loss rates of the uncoated and coated SEDYs could be observed at day 6. After that, the difference was gradually narrowed. It might be due to the unique dense network structure and hydrophobicity of WPNFs, which hindered the loss of moisture. Moreover, since CA was a fat-soluble substance, the WPNFs film containing CA restricted the flow of water molecules. As a result, WPNFs–CA/Gly film slowed the diffusion rate of water molecules and reduced the weight loss of SEDYs.

Figure 9b showed an actual picture of the SEDYs salted duck egg yolks after 10 days of storage, including uncoated SEDYs, SEDYs coated with WPI/Gly, and WPNFs–CA/Gly coating. The SEDYs of the control group (uncoated) and SEDYs coated with WPI–CA/Gly coating had different degrees of cracks. On the contrary, the surface of SEDYs coated with WPNFs–CA/Gly coating was smoother. Furthermore, with the increase of storage days, the weight loss rate of SEDYs increased approximately linearly, and the increase rate was fast first and then slow. The most obvious difference between the weight loss rate of SEDYs coated with WPNFs–CA/Gly coating and the other two groups was observed on the sixth day. The weight loss rate of yolks coated with WPNFs–CA/Gly was only 3.34% compared with the control groups. It could be concluded that the WPNFs–CA/Gly coating can effectively reduce water loss in coated food.

#### 3.7.2. Texture Property

The texture property of non-coated and coated SEDYs during storage is presented in Table 2. It was observed that the SEDYs themselves possessed certain hardness. This was because, during the salting process of duck eggs, the salt had penetrated into the eggs. A part of the water in the egg yolk was forced out, causing the fat to accumulate, which in turn harden the egg yolk. Over time, the hardness of the egg yolks reached its maximum (547.90 ± 3.80 N). In contrast, the hardness of the coated egg yolks was improved after 10 days of storage. The hardness increase rate of egg yolks coated with the WPI–CA/Gly coating was 28.01%. It was noted that the egg yolks coated with the WPNFs–CA/Gly coating had the lowest hardness increase rate (18.22%). Since the surface of the WPNFs–CA/Gly film is flat and free of pores (as shown in Figure 8), this hindered the loss of moisture. The overall sensory score of egg yolk (hardness, springiness, chewiness) decreased with time, and this was probably due to a combination of water loss and lipid decomposition.

## 4. Conclusions

In this study, WPNFs could be self-assembled by WPI at high temperature (80 °C) incubation for 10 h with constant magnetic stirring. The WPNFs–CA emulsion was prepared by high-pressure homogenization of WPNFs and CA. Results from CLSM proved that WPNFs–CA emulsions had smaller particle size and a better distribution than WPI–CA emulsions. The WPNFs–CA/Gly coating prepared from this WPNFs–CA emulsion had a higher antibacterial activity. The WPNFs–CA/Gly films have smooth and continuous surfaces and best transmittance. SDEYs coated with the WPNFs–CA/Gly coating were superior to salted egg yolk treated with the WPI–CA/Gly coating, showing the lowest weight loss rate. Textural properties were significantly improved by the WPNFs–CA/Gly coating on SDEYs. It was noted that the egg yolks coated with the WPNFs–CA/Gly coating had the lowest hardness increase rate (18.22%). As the results mention above, the edible coatings prepared with functionalized WPNFs can be considered a diverse tool for the long-term preservation of food. Hence, WPNFs-based coatings may have a good development prospect in the food industry.

## Figures and Tables

**Table 1:** Thickness, transmittance, color values of edible films.

Films	Thickness (mm)	Transmittance (%)	Color Values		
			L*	a*	b*
WPI/Gly	0.184 ± 0.066 ^a^	46.7 ± 1.3 ^bc^	57.13 ± 1.84^c^	3.57 ± 0.88 ^a^	2.64 ± 0.58 ^b^
WPNFs/Gly	0.182 ± 0.034 ^a^	49.2 ± 1.4 ^c^	63.00 ± 0.16 ^d^	2.29 ± 0.81 ^c^	1.50 ± 0.14 ^a^
WPI–CA/Gly	0.232 ± 0.045 ^b^	41.5 ± 0.1 ^a^	39.87 ± 0.24 ^a^	1.20 ± 0.36 ^b^	12.82 ± 0.42 ^c^
WPNFs–CA/Gly	0.216 ± 0.038 ^ab^	45.7 ± 2.1 ^b^	46.89 ± 0.18 ^b^	2.94 ± 1.07 ^c^	10.00 ± 1.16 ^c^

^a,b,c,d^ Different letters mean significant difference at *p* < 0.05 in same column. L*-(lightness/brightness), a*-(redness/greenness), and b*-(yellowness/blueness) values.

**Table 2:** Texture profile analysis of coated SEDYs during storage at 4 °C.

-	Hardness (N)		Springiness (Mm)		Chewiness (N/m)	
-	0 day	10 days	0 day	10 days	0 day	10 days
Uncoated	327.37 ± 7.73 ^a^	547.90 ± 3.80 ^c^	0.79 ± 0.02 ^b^	0.37 ± 0.06 ^a^	150.48 ± 1.76 ^b^	368.01 ± 7.37 ^c^
WPI–CA /Gly	343.81 ± 3.11 ^b^	477.63 ± 1.37 ^b^	0.73 ± 0.01 ^a^	0.57 ± 0.06 ^b^	115.77 ± 6.68 ^a^	342.88 ± 5.11 ^b^
WPNFs–CA/Gly	341.5 ± 0.70a ^b^	417.97 ± 2.46 ^a^	0.83 ± 0.02 ^b^	0.64 ± 0.05 ^b^	124.5 ± 0.99 ^b^	320.00 ± 2.69 ^a^

^a,b,c^ Different letters mean significant difference at *p* < 0.05 in same column.

## Abbreviations

SDEY	salted duck egg yolk
WPNFs	whey protein isolate nanofibers
Gly	glycerol
WPI	whey protein isolate
CA	carvacrol
EC	edible coating
EFs	edible films
TEM	transmission electron microscopy
SDS-PAGE	sodium dodecyl sulfate-polyacrylamide gel electrophoresis
SEM	scanning electron microscopy
CLSM	confocal laser scanning microscopy
